# Quantitative evaluation of fibrosis in idiopathic orbital inflammatory pseudotumor by IVIM-DWI

**DOI:** 10.3389/fopht.2025.1545761

**Published:** 2025-05-26

**Authors:** Jian Pu, Xing Xu, Peng Wang, Wei Su, Juwei Shao, Yinhua Yang, Mingqin Li, Jianbo Li

**Affiliations:** ^1^ Department of Radiology, Affiliated Hospital of Yunnan University, Kunming, China; ^2^ Department of Radiology, 920 Hospital of the joint service support force of the Chinese people’s Liberation Army, Kunming, China; ^3^ School of Clinical Medicine, Dali University, Dali, China

**Keywords:** orbital cavity, inflammatory pseudotumor, magnetic resonance imaging, incoherent motion in voxels, pathology, fibrosis

## Abstract

**Objective:**

To quantitatively evaluate the degree of fibrosis in Idiopathic Orbital Inflammatory Pseudotumor (IOIP) using Intravoxel Incoherent Motion Diffusion-Weighted Imaging (IVIM-DWI).

**Methods:**

Twenty eight patients (32 eyes) with idiopathic orbital inflammatory pseudotumor were pathologically diagnosed in the Affiliated Hospital of Yunnan University from August 2019 to August 2024. Routine orbital MRI plain scan and ivim-dwi scan were completed before surgery, and the true diffusion coefficient (d), pseudo diffusion coefficient (d*) and perfusion fraction (f) were obtained. According to the proportion of chronic inflammatory cells and fibrous tissue components in postoperative histopathological sections, it was defined that fibrous cells accounted for less than 10% of the total number of cells as negative (–), and > 10% as positive (+), of which 10%%25% were +, 25%%50% were + +, 50%%75% were + + +, and > 75% were + + +. Taking the proportion of fibrocytes in histopathological sections as the standard of fibrosis degree, the correlation between ivim-dwi parameters and the proportion of fibrocytes in pathological sections was analyzed. Data were analyzed by SPSS 19.0 statistical software package, and P < 0.05 was considered statistically significant.

**Results:**

Among 28 cases (32 eyes) with idiopathic orbital inflammatory pseudotumor, there was no statistical difference between gender, age and the degree of IOIP fibrosis (P > 0.05). There was a statistically significant difference between the proportion of IOIP fiber composition and the D value and F value in IVIM-DWI parameters (P < 0.05), and the sensitivity of F value was higher than D value in the comparison of the two parameters combined with ROC curve analysis; There was no statistical difference between the D * value and the degree of fibrosis (P > 0.05).

**Conclusion:**

The D value and F value of IVIM-DWI showed a correlation with the proportion of IOIP fiber components, and the sensitivity of F value was higher than D value. Ivim-dwi examination parameters can be used as quantitative and objective indicators to evaluate the degree of fibrosis of lesions before IOIP.

## Highlights

- The degree of fibrosis in IOIP is closely related to the treatment regimen.
- IVIM-DWI is a quantitative, noninvasive and radiation free examination method.
- The D value and f value of IVIM-DWI can evaluate the degree of IOIP fibrosis preoperatively.

## Introduction

Idiopathic orbital inflammatory pseudotumor (IOIP) is a primary autoimmune disease in the orbit, accounting for 5% - 7.5% of orbital lesions (1), and the incidence ranks third in orbital lesions (2). The current treatment methods include hormone therapy, surgical resection therapy and radiological treatment (3, 4). The treatment methods are mainly based on different histopathological components, which mainly include chronic inflammatory cell infiltration (mainly lymphocytes) and fibrous tissue hyperplasia. The tissue components are different, and the treatment methods and prognosis are quite different. IOIP with inflammatory cell infiltration is sensitive to hormone therapy and has a good prognosis. IOIP with fibrous tissue hyperplasia has poor effect on hormone therapy and high recurrence rate (5, 6). Radiotherapy and surgical resection are the first choice. Improper or no treatment of IOIP can lead to the destruction of intraorbital structure due to the excessive proliferation of intraorbital fibrous tissue in patients, which eventually leads to irreversible visual impairment and even requires surgical enucleation of the eyeball (7). Therefore, preoperative evaluation of IOIP tissue composition and fibrosis is essential for the selection of clinical treatment options and the prognosis of patients.

Histopathology is the gold standard for IOIP diagnosis and tissue composition analysis (8). It needs to be obtained by biopsy or surgical resection of diseased tissue. It is a traumatic and invasive examination method (5). Improper operation may lead to intraorbital soft tissue injury such as ocular muscle and optic nerve. Therefore, it is urgent to seek an alternative non-invasive diagnosis method. MRI examination has the characteristics of high tissue resolution, noninvasive and non-radiation, and is widely used in the diagnosis and treatment evaluation of orbital diseases (9). In recent years, the application of MRI in the diagnosis of IOIP has been routinely carried out in clinical work (10), and various fMRI applications in the diagnosis of IOIP and other diseases have become a research hotspot (11–13). Intravoxel incoherent motion diffusion-weighted imaging (IVIM-DWI) is a functional imaging technique of magnetic resonance examination (14). Based on the biexponential model, multiple parameters can be obtained: true diffusion coefficient (d), pseudo diffusion coefficient (D*), perfusion fraction (f), which can comprehensively reflect the quantitative parameters of water molecule movement in tissue cells and blood microcirculation, without the interference of water molecule diffusion movement outside tissue cells. IVIM has high diagnostic and differential diagnostic value in liver, brain tumor, kidney and other diseases (15–19). The previous research results revealed that the D value and F value of IVIM-DWI parameters are correlated with the histopathological classification of IOIP, which can be used as a good index for preoperative evaluation of the pathological classification of IOIP (20). Based on the previous research results, this study applied IVIM-DWI examination technology to further quantitatively analyze the correlation between IVIM-DWI parameters and the content of fibrous tissue in IOIP lesions according to the proportion of fibrous components in pathological sections. The purpose is to determine the content of fibrous components in the lesions preoperatively and provide the basis for the preoperative personalized treatment plan of IOIP patients.

## Materials and methods

### Patients

**Figure 1:**
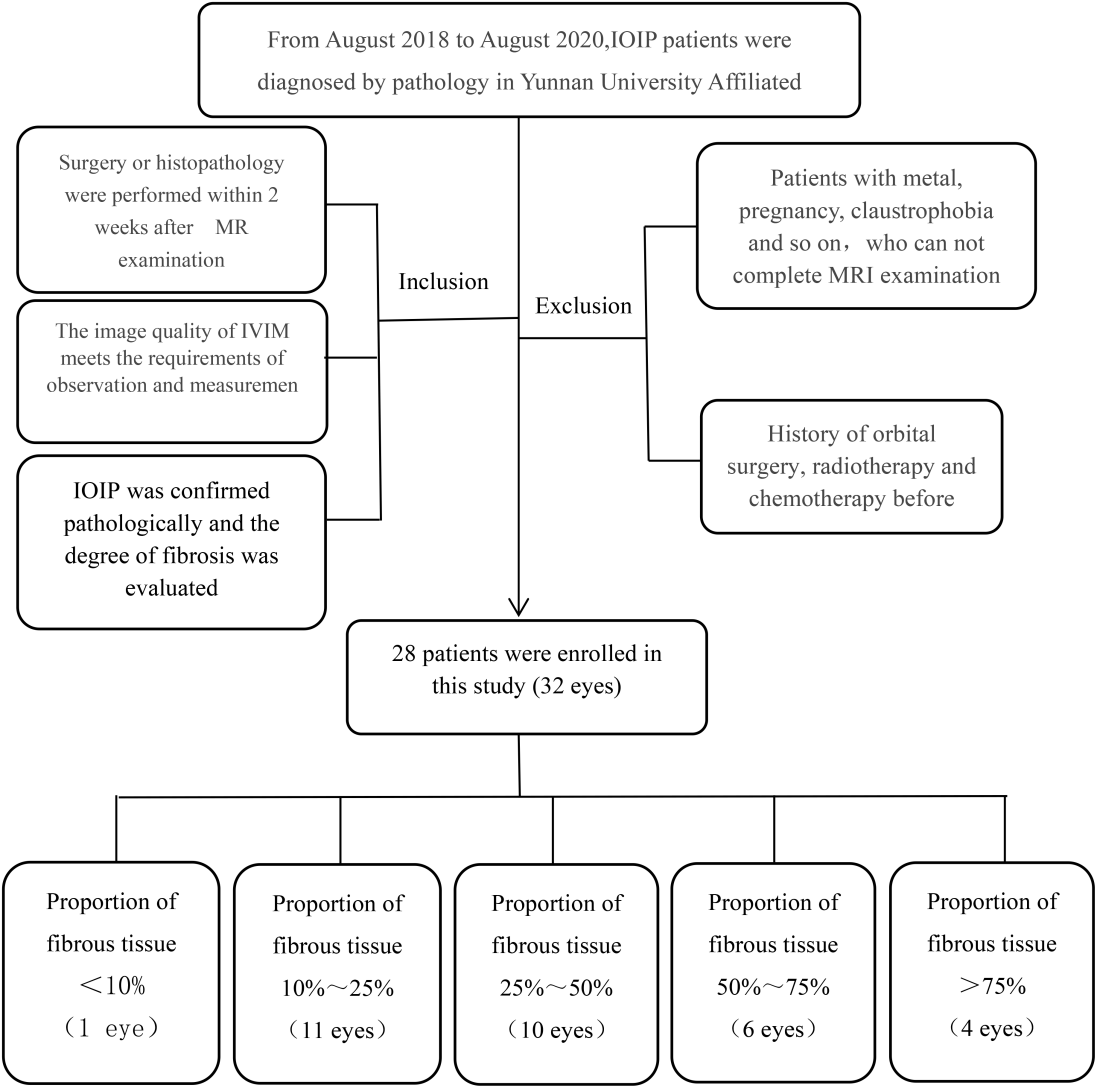
Flowchart shows the patient recruitment in this study.

This study was approved by the review board of the Affiliated Hospital of Yunnan University, and the requirement of informed consent was waived because the study was retrospective. Patients with pathologically diagnosed idiopathic orbital inflammatory pseudotumor from August 2019 to August 2024 in the Affiliated Hospital of Yunnan University were included as the research objects. All cases completed MRI plain scan and ivim-dwi scan before clinical intervention, and underwent biopsy or surgical resection for pathological examination within 2 weeks after MRI examination. According to the pathological results, patients diagnosed with IOIP were screened and their pathological sections were analyzed for the proportion of fiber components. The exclusion criteria were those who underwent orbital tumor surgery, radiotherapy and chemotherapy before the examination, those who did not complete the ivim-dwi examination before the operation, or those who lacked pathological examination results. Finally, 28 patients (32 eyes) were included in the study (**Figure 1**).

### Image data acquisition and processing

#### MRI scan parameters

Conventional MRI and IVIM-DWI were performed with a GE signal 1.5T HDX superconducting MR scanner (8-channel head coil). Plain scan sequences included cross-sectional T2WI,T1WI, fat suppression T2WI, coronal T2WI, single-index DWI, and double-index IVIM. The flat scan matrix was 224 ×160, repetition time (TR) was 3,000 ms, echo time (TE) was 90ms, field of view (FOV) was 24 × 24 cm, number of excitations(NEX) was l, layer thickness was 4 mm, layer spacing was 1 mm, the number of layers was 14, and the turning angle was 15°.Single-shot spin echo planar imaging was used for DWI scanning, with b value of 1,000 s/mm2 (TR: 3,500 ms, TE: 75ms), matrix of 256 × 256, and FOV of 18 × 18 cm. For axial IVIM, the parameters were as follows: b values 0, 20, 50, 75, 100,150, 200, 400, 800, 1,000, 1,200, 1,500, and 2,000 s/mm2, TR 5,098 ms, TE 69 ms, layer thickness 4 mm, interval 1 mm, matrix140 × 125, FOV 24 × 24 cm, NEX 1, and number of layers = 20.

#### Postprocessing of MRI image

**Figure 2:**
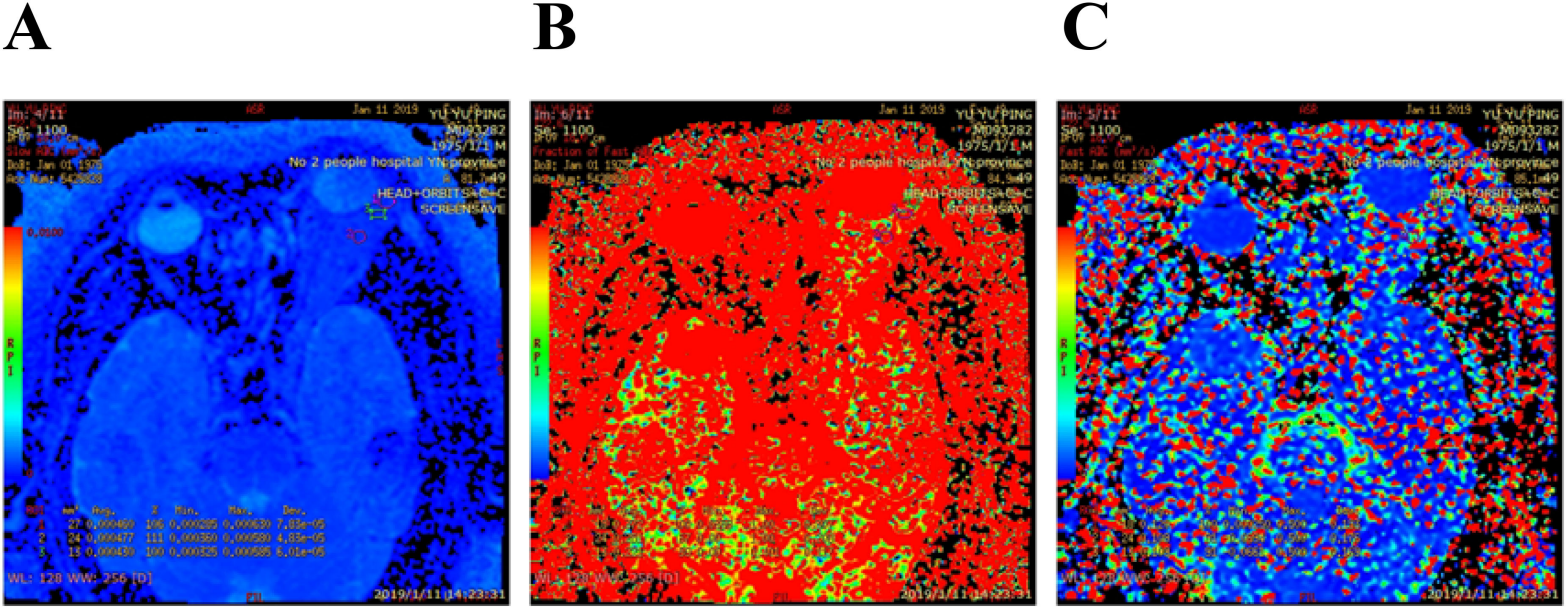
Idiopathic orbital inflammatory pseudotumor of left lacrimal gland, Pseudocolor IVIM image: **(A)** D value = 0.456 × 10^−3^ mm2/s; **(B)** D * value = 64.9 × 10^−3^ mm2/s; **(C)** f value = 28.1%.

The MR multisequence plain scan and IVIM sequence scan data were collected. The acquired images were analyzed and postprocessed by two imaging experts on ADW workstation READY view software. The region of interest (ROI) was defined as the most uniform area of the abnormal signal area of the lesion, selected manually to determine the scope of the lesion and to avoid the cystic area and necrotic area. The ROI area was required to be smaller than the whole lesion, and an average value was taken from three measurements. The ROI selected in each case therefore varied according to the size of the measured object, with areas ranging from 8 to 10 mm2. The ROI selected by the image measurement is the pathological sample region. The parameters D, D*, and f were measured using inclined view software (**Figure 2**).

#### Interpretation of pathological section results

**Figure 3:**
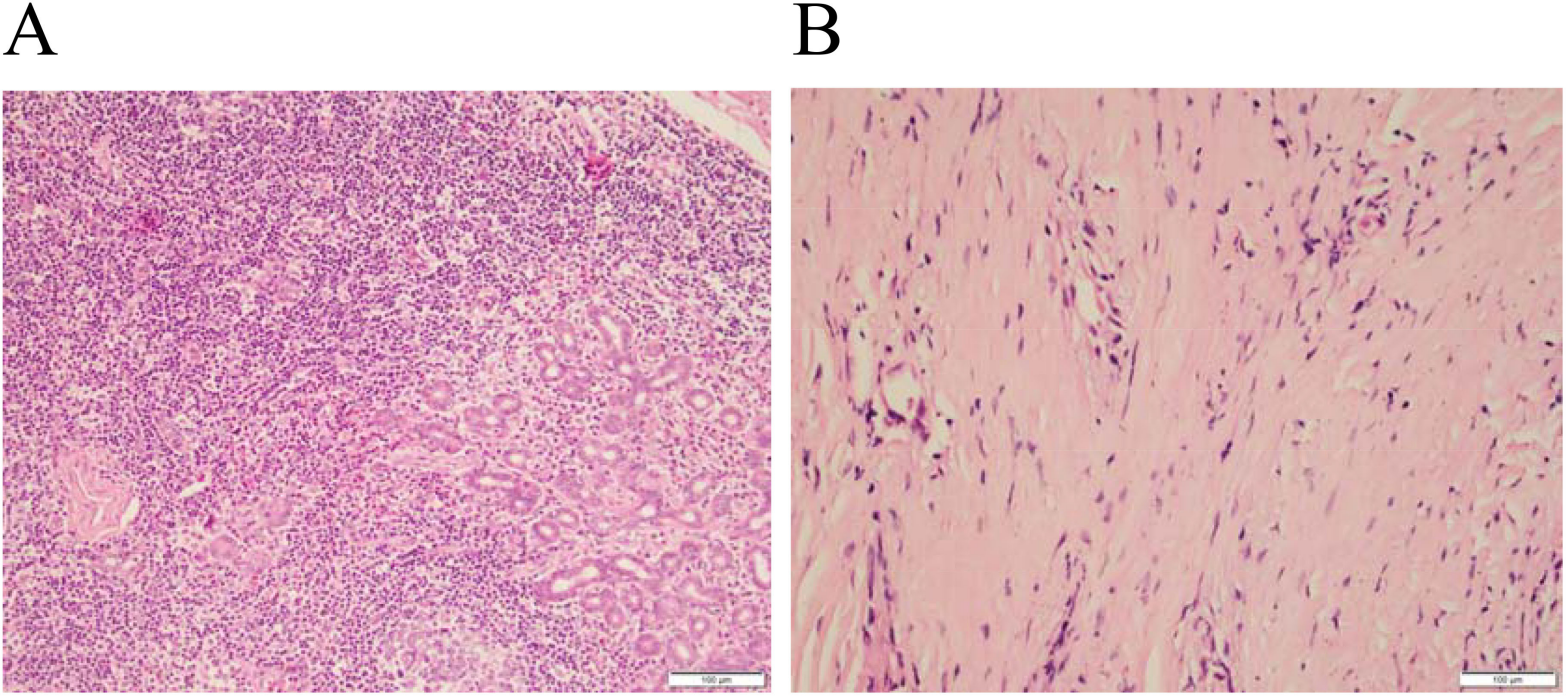
Idiopathic orbital inflammatory pseudotumor. Pathological sections HE staining×400 times showed: **(A)** Proportion of fibrocytes 10%%25%(+), **(B)** Proportion of fibrocytes >75%(+ + + +).

Histopathological sections were obtained after surgery, and hematoxylin eosin staining was used to observe the ratio of chronic inflammatory cells to fibrous tissue components under a light microscope (**Figure 3**). According to the proportion of fibrocytes in the total number of cells, the degree of fibrosis was divided into negative (–) and positive (+), of which 10% 25% were +, 25% 50% were + +, 50% 75% were + + +, and > 75% were + + + +.

### Statistical methods

SPSS 17.0 statistical software package was used to analyze the data. In general data, age was expressed as mean ± standard deviation, and gender was tested by Pearson chi square. Pearson correlation analysis was used to analyze the consistency of the measured data of the two experts, and the correlation coefficient > 0.4 was relatively consistent. The correlation between D value, D* value and F value of IVIM parameters and pathological classification was analyzed by ANOVA, P < 0.05 had statistical difference. ROC curves were drawn to compare the sensitivity and specificity of IVIM parameters D value and f value in evaluating the degree of IOIP fibrosis. P < 0.05 was considered statistically significant.

## Results

### Patients’ clinical data

Among the 28 patients (32 eyes), there were 15 males and 13 females, all of whom were of Han nationality, aged from 23 to 75 years, with an average age of 52.18 ± 15.62 years. Among them, 1 case (1 eye) with fibrocytes accounting for less than 10% of the total cells was female, aged 44 years; There were 11 cases (11 eyes) with 10%%25% (+), 4 males and 7 females, aged from 39 to 75 years, with an average age of 57.81 ± 13.01 years; There were 10 eyes (10 eyes) with 25%%50% (+ +), 6 males and 4 females, aged from 35 to 71 years, with an average age of 58.50 ± 8.18 years; There were 6 eyes with 50%%75% (+ +), 5 males and 2 females, aged from 23 to 75 years, with an average age of 47.67 ± 20.94 years 75% (+ + +) 4 eyes, 3 females and 1 male, aged from 23 to 35 years, with an average age of 29.50 ± 4.93 years. Gender and age had no correlation with the degree of IOIP fibrosis (P > 0.05) (**Table 1**).

**Table 1:** Clinical data and proportion of fibrocytes in 28 patients (32 eyes) with IOIP.

General information	Proportion of fibrocytes(%)					F value	P value
	-	+	++	+++	++++		
	(n=1)	(n=11)	(n=10)	(n=6)	(n=4)		
Age	44	57.81±13.01	58.50±8.18	47.67±20.94	29.50±4.93	2.518	0.077
Gender						0.648	0.427
Male	0	4	6	5	1		
Female	1	7	4	2	3		

### Correlation between D value, D* value and f value and the percentage of fibrocytes In IOIP pathological sections

In the IOIP pathological section, the D value of the percentage of fibrocytes in all cells < 10% (–) was 0.507 × 10^-3^mm2/s,D* was 0.479 × 10^-3^mm2/s, and f value was 14.600%; The D value of the percentage of fibrocytes accounting for 10%%25% (+) of all cells was (0.473 ± 0.337) × 10^-3^mm2/s, D* was (58.118 ± 30.821) × 10^-3^mm2/s, and f value was (25.118 ± 24.095)%; The D value of 25%%50% (+ +) of fibrocytes was (0.524 ± 0.295) × 10^-3^mm2/s, D* was (57.860 ± 16.419) × 10^-3^mm2/s, f value was (32.460 ± 35.911)%; The D value of fiber cells accounting for 50%%75% (+ +) of all cells was (0.547 ± 0.852) × 10^-3^mm2/s, D* was (32.508 ± 29.346) × 10^-3^mm2/s, and f value was (40.417 ± 16.975)%; The D value of fibrocytes accounting for > 75% (+ + +) of all cells was (0.632 ± 0.361) × 10^-3^mm2/s, D* was (24.412 ± 22.483) × 10^-3^mm2/s, and f value was (43.925 ± 12.842)%. f value is the largest value in the evaluation of the proportion of fibrocytes in IOIP pathological sections, followed by D value, which is a sensitive indicator of the degree of fibrosis in IOIP pathological sections (P < 0.05). There is no correlation between D* value and IOIP fibrosis (P > 0.05) (**Table 2**).

**Table 2:** Correlation between percentage of fibrocytes and IVIM parameters in 28 patients (32 eyes) with IOIP.

IVIM parameter	Percentage of fibrocytes in IOIP pathological sections					F value	P value
	-	+	++	+++	++++		
D(×10-3mm2/s)	0.507	0.473±0.337	0.524±0.295	0.547±0.852	0.632±0.361	8.920	<0.05
D*(×10-3mm2/s)	0.479	58.118±30.821	57.860±16.419	32.508±29.346	24.412±22.483	2.186	>0.05
f(%)	14.600	25.118±24.095	32.460±35.911	40.417±16.975	43.925±12.842	63.383	<0.05

### The efficacy of D value and F value in the evaluation of IOIP fibrosis

**Figure 4:**
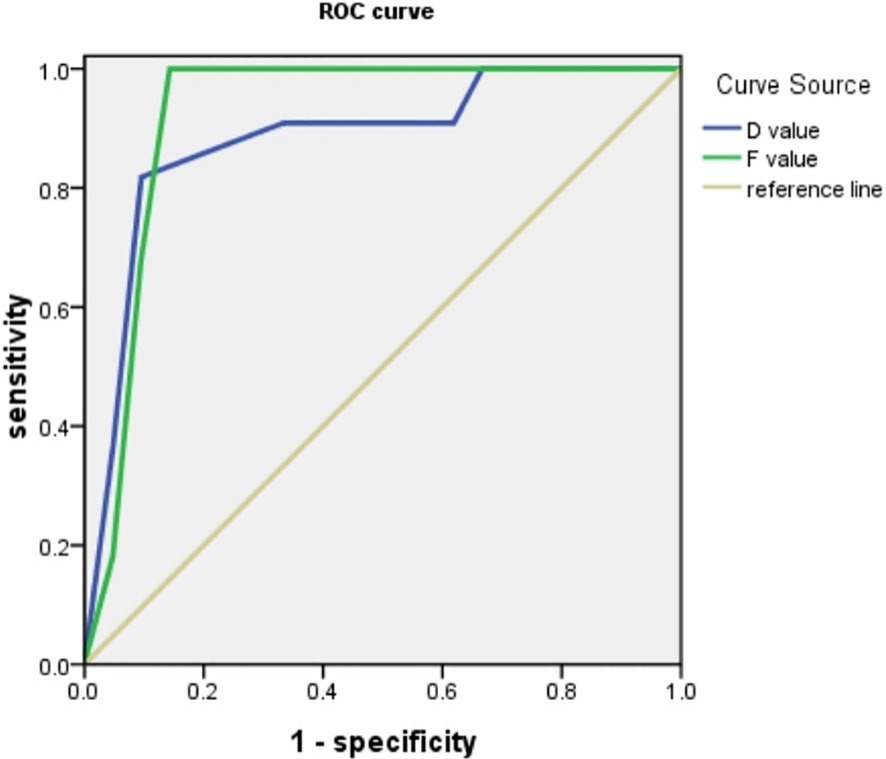
ROC curve showed that both D value and F value could quantitatively evaluate the degree of IOIP fibrosis, and the area under the curve of F value was higher than D value.

The ROC curve analysis results showed that (**Figure 4**, **Table 3**), the area under the ROC curve of D value was 0.933, and the specificity and sensitivity of quantitative evaluation of IOIP fibrosis were 81.8% and 95%. The area under the ROC curve of f value was 0.890, and the specificity and sensitivity of quantitative evaluation of IOIP fibrosis were 100% and 95%. The f value of IVIM has the highest sensitivity for quantitative evaluation of IOIP fibrosis.

**Table 3:** Comparison of IVIM parameters in the diagnosis of IOIP fibrosis.

	AUC	P value	Sensitivity	Specificity
D value	0.922	<0.05	81.8%	95%
f value	0.890	<0.05	100%	95%

## Discussion

Idiopathic inflammatory pseudotumor of the orbit is a non-specific inflammatory disease of the orbit with unknown cause, which is mainly characterized by inflammation, edema, fibrosis and lymphocyte infiltration in the orbital tissue (21). The degree of fibrosis is one of the important factors affecting the prognosis of IOIP patients (20). Therefore, accurate preoperative evaluation of the degree of fibrosis is of great significance for guiding clinical treatment. However, traditional imaging methods have limitations in evaluating the degree of fibrosis in IOIP (22), making it difficult to accurately quantify the fiber composition. IVIM-DWI is a magnetic resonance functional imaging technology, which can provide more detailed microscopic information by measuring the diffusion and perfusion properties of water molecules in tissues, and provide a new means for evaluating the degree of fibrosis of IOIP (23).

The results of this study showed that the IVIM-DWI parameters D and f values were correlated with the proportion of IOIP fiber composition, while the D* value was not correlated with the proportion of fiber composition. The D value reflects the true diffusion characteristics of water molecules in tissues, depending on the restricted diffusion of water molecules inside and outside cells caused by inflammatory cell infiltration, macromolecules and fibrosis. The increased degree of fibrosis in IOIP leads to the restricted diffusion of water molecules in tissues, so the D value decreases. This result is consistent with previous studies in other fibrotic diseases (24–26), and also verifies the potential value of D value in evaluating the degree of fibrosis. The f value reflects the tissue micro perfusion and the local vascular distribution characteristics of the tissue. Its value is positively correlated with the number of capillaries in the tissue (27, 28). The increase of fibrocytes in IOIP lesions leads to vascular compression and lumen stenosis, so the tissue micro perfusion is reduced, and the f value is reduced. This study showed that there was no statistical difference between the D* value and the degree of IOIP fibrosis. This result is consistent with the result revealed by our previous study (20) that there is no correlation between the parameter D* value of IVIM-DWI and IOIP classification. D* is a pseudo diffusion coefficient, which mainly reflects the information of blood microfluidics in local tissues. Its value is greatly affected by the number and size of the selected b value (29). In this study, D* value is not correlated with the degree of IOIP fibrosis, which may be related to this reason, as well as the complex and variable blood microfluidics in IOIP lesions (30). Therefore, D* value may not be a reliable indicator when evaluating the degree of IOIP fibrosis.

However, in this study, the correlation between F value and the proportion of fiber components was higher, and ROC curve analysis showed that the sensitivity of f value was higher than D value, which suggested that f value may have higher value in evaluating the degree of fibrosis in IOIP. Based on the fact that the D value mainly reflects the restricted diffusion and movement of intercellular water molecules, while the f value mainly reflects the characteristics of microcirculation perfusion of intercellular capillaries, we speculate that the more abundant fiber components in IOIP lesions, the less intercellular water molecules, and the more abundant capillaries may be, and the degree of vascular compression is more significant. Therefore, f value has higher sensitivity and specificity in evaluating the degree of fibrosis of IOIP, and it is a better indicator for evaluating the degree of fibrosis.

### There were some limitations in this study that should be mentioned

First, the sample size of this study is limited, and the number of cases will continue to be expanded in the future. Secondly, image quality is affected by motion artifacts, and parameter measurement is greatly affected by scanning parameters and image post-processing methods.

## Conclusion

The D and F values of IVIM-DWI were correlated with the proportion of IOIP fiber components, indicating that IVIM-DWI examination method can be used as a quantitative and objective index to evaluate the degree of fibrosis of lesions before IOIP. Among them, the parameter f value shows higher sensitivity and is a better indicator to evaluate the degree of fibrosis.

## Data Availability

The raw data supporting the conclusions of this article will be made available by the authors, without undue reservation.
